# Supplementary material for: Antibody binding reports spatial heterogeneities in cell membrane organization
Source: Nat Commun. 2023 May 20;14:2884. doi: 10.1038/s41467-023-38525-2 (PMC10199101; doi:10.1038/s41467-023-38525-2)
Supplement: Supplementary file 3 — Description of Additional Supplementary Files [file 41467_2023_38525_MOESM3_ESM.pdf]

File name: Supplementary Movie 1

Description: MD simulation of FITC sensor (green) with 64 linker monomer units (blue), each of diameter 0.33 nm. Sensors were tethered to the bottom of the simulation box at a surface density of 1000/μm^2.

File name: Supplementary Movie 2

Description: MD simulations of antibodies in monodisperse PEG surface. MD simulation of ideal gas antibodies (red) of diameter 4 nm and N = 45 PEG brush (gray) with monomer diameter 0.33 nm and surface density of 30000/μm^2.

File name: Supplementary Movie 3

Description: MD simulation of antibodies in bidisperse red blood cell surface. MD simulation of ideal gas antibodies (red) of diameter 11 nm in the presence of a bidisperse brush. Glycophorin A (dark green) is represented as a seven-bead chain with monomer diameter 2 nm and surface density of 1300/µm2, while Band 3 (light green) is represented as a 10 bead chain with monomer diameter 1 nm and surface density of 6700/μm^2.
