# Supplementary material for: Antibody binding reports spatial heterogeneities in cell membrane organization
Source: Nat Commun. 2023 May 20;14:2884. doi: 10.1038/s41467-023-38525-2 (PMC10199101; doi:10.1038/s41467-023-38525-2)
Supplement: Supplementary file 7 — Reporting Summary [file 41467_2023_38525_MOESM7_ESM.pdf]

Corresponding author(s): Sho Takatori

Last updated by author(s): Apr 12, 2023

Reporting Summary

Nature Portfolio wishes to improve the reproducibility of the work that we publish. This form provides structure for consistency and transparency in reporting. For further information on Nature Portfolio policies, see our Editorial Policies and the Editorial Policy Checklist.

Statistics

For all statistical analyses, confirm that the following items are present in the figure legend, table legend, main text, or Methods section.

n/a	Confirmed	
☐	☒	The exact sample size (*n*) for each experimental group/condition, given as a discrete number and unit of measurement
☐	☒	A statement on whether measurements were taken from distinct samples or whether the same sample was measured repeatedly
☒	☐	The statistical test(s) used AND whether they are one- or two-sided<br>*Only common tests should be described solely by name; describe more complex techniques in the Methods section.*
☒	☐	A description of all covariates tested
☒	☐	A description of any assumptions or corrections, such as tests of normality and adjustment for multiple comparisons
☐	☒	A full description of the statistical parameters including central tendency (e.g. means) or other basic estimates (e.g. regression coefficient) AND variation (e.g. standard deviation) or associated estimates of uncertainty (e.g. confidence intervals)
☒	☐	For null hypothesis testing, the test statistic (e.g. *F*, *t*, *r*) with confidence intervals, effect sizes, degrees of freedom and *P* value noted<br>*Give P values as exact values whenever suitable.*
☒	☐	For Bayesian analysis, information on the choice of priors and Markov chain Monte Carlo settings
☒	☐	For hierarchical and complex designs, identification of the appropriate level for tests and full reporting of outcomes
☒	☐	Estimates of effect sizes (e.g. Cohen's *d*, Pearson's *r*), indicating how they were calculated

Our web collection on statistics for biologists contains articles on many of the points above.

Software and code

Policy information about availability of computer code

Data collection	Micromanager v1.4<br>HOOMD v2.9.3 available at https://github.com/glotzerlab/hoomd-blue
Data analysis	MATLAB (v2020b)<br>Cell surface optical profilometry MATLAB code available at https://github.com/smson-ucb/CSOP

For manuscripts utilizing custom algorithms or software that are central to the research but not yet described in published literature, software must be made available to editors and reviewers. We strongly encourage code deposition in a community repository (e.g. GitHub). See the Nature Portfolio guidelines for submitting code & software for further information.

Data

Policy information about availability of data

All manuscripts must include a data availability statement. This statement should provide the following information, where applicable:

- Accession codes, unique identifiers, or web links for publicly available datasets
- A description of any restrictions on data availability
- For clinical datasets or third party data, please ensure that the statement adheres to our policy

All data generated or analyzed during this study are included in this published article (and its supplementary information files).

Human research participants

Policy information about studies involving human research participants and Sex and Gender in Research.

Reporting on sex and gender	*Use the terms sex (biological attribute) and gender (shaped by social and cultural circumstances) carefully in order to avoid confusing both terms. Indicate if findings apply to only one sex or gender; describe whether sex and gender were considered in study design whether sex and/or gender was determined based on self-reporting or assigned and methods used. Provide in the source data disaggregated sex and gender data where this information has been collected, and consent has been obtained for sharing of individual-level data; provide overall numbers in this Reporting Summary. Please state if this information has not been collected. Report sex- and gender-based analyses where performed, justify reasons for lack of sex- and gender-based analysis.*
Population characteristics	*Describe the covariate-relevant population characteristics of the human research participants (e.g. age, genotypic information, past and current diagnosis and treatment categories). If you filled out the behavioural & social sciences study design questions and have nothing to add here, write "See above."*
Recruitment	*Describe how participants were recruited. Outline any potential self-selection bias or other biases that may be present and how these are likely to impact results.*
Ethics oversight	*Identify the organization(s) that approved the study protocol.*

Note that full information on the approval of the study protocol must also be provided in the manuscript.

Field-specific reporting

Please select the one below that is the best fit for your research. If you are not sure, read the appropriate sections before making your selection.

☒ Life sciences ☐ Behavioural & social sciences ☐ Ecological, evolutionary & environmental sciences

For a reference copy of the document with all sections, see nature.com/documents/nr-reporting-summary-flat.pdf

Life sciences study design

All studies must disclose on these points even when the disclosure is negative.

Sample size	No statistical tests were applied to determine sample size. In experiments on reconstituted supported lipid bilayers and red blood cells, sample sizes of greater than 50 beads or cells per bulk antibody concentration were used to fit each binding isotherm. In experiments using mammalian cells and giant unilamellar vesicles, greater than 15 cells or vesicles per antibody bulk concentration were analyzed to fit each binding isotherm. In all experiments, 6-10 bulk concentrations were measured per binding isotherm, giving total sample sizes of greater than 90-500.<br>In molecular dynamics simulations, polymer chain configurations were collected across 100 snapshots in 15 realizations to produce spatial sensor distributions, yielding sample sizes of 15000. In simulations of synthetic and red blood cell surfaces, greater than 2000 polymer chains and 1000 antibodies were simulated in each system.
Data exclusions	No data were excluded from the analysis. Obviously-lysed cells containing fluorophore on the inside as well as on the membrane were not imaged.
Replication	We averaged and collected statistics over greater than 15-50 cells or beads per IgG bulk concentration, with 6-10 bulk concentrations per binding isotherm measurement. We collected replicate measurements for a subset of cholesterol-PEG-FITC sensors on beads and red blood cells, as well as on T47D cells, confirming the reported dissociation constants.
Randomization	Not applicable as covariate grouping was not used.
Blinding	Blinding was not applicable, as there were no human or animal subjects involved. Quantitative measurements, as described in methods, did not require subjective decision-making on the part of the researchers. Random microscope fields of view were chosen and all visible cells or beads within that field of view were analyzed.

Reporting for specific materials, systems and methods

We require information from authors about some types of materials, experimental systems and methods used in many studies. Here, indicate whether each material, system or method listed is relevant to your study. If you are not sure if a list item applies to your research, read the appropriate section before selecting a response.

Materials & experimental systems

n/a	Involved in the study
☐	☒ Antibodies
☐	☒ Eukaryotic cell lines
☒	☐ Palaeontology and archaeology
☒	☐ Animals and other organisms
☒	☐ Clinical data
☒	☐ Dual use research of concern

Methods

n/a	Involved in the study
☒	☐ ChIP-seq
☒	☐ Flow cytometry
☒	☐ MRI-based neuroimaging

Antibodies

Antibodies used

1. Fluorescein monoclonal antibody (clone 1F8-1E4) Invitrogen (catalog #31242).
2. Biotin Alexa Fluor 647-tagged monoclonal antibody (clone BK1/39) Santa Cruz Biotechnology (catalog #sc-53179 AF647).
3. Anti-mouse CD45RB Alexa Fluor® 647 rat monoclonal antibody (clone #C363-16A) Biolegend (catalog #10331).
4. Anti-mouse CD45RB Alexa Fluor® 647 rat monoclonal antibody (clone #I3/2.3) Biolegend (catalog #147715).
5. Cholera toxin beta mouse monoclonal antibody (clone #23043) Novus Biologicals (catalog #NB100-64675).

Validation

1. Fluorescein monoclonal antibody was verified on custom-made cholesterol-PEG0.5k-FITC constructs bound to supported bilayers. Antibody was also verified on FITC-conjugated beads by Badgujar, et al., PLOS 2020.
2. Biotin Alexa Fluor 647-tagged monoclonal antibody was verified on biotin-conjugated lipids in supported lipid bilayers. Antibody was previously verified by Chan, K., et al. eLife, 2020.
3. Anti-mouse CD45RB Alexa Fluor® 647 rat monoclonal antibody (clone #C363-16A) was verified by Son, S., et al. PNAS 2020.
4. Anti-mouse CD45RB Alexa Fluor® 647 rat monoclonal antibody (clone #I3/2.3) was verified by Son, S., et al. PNAS 2020.
5. Cholera toxin beta mouse monoclonal antibody was verified on Cholera Toxin Subunit B (Recombinant), Alexa Fluor 488 Conjugate (Invitrogen C34775) bound to GM1 on a supported lipid bilayer.

Eukaryotic cell lines

Policy information about cell lines and Sex and Gender in Research

Cell line source(s)

1. HeLa human cervical cancer cells (ATCC CCL-2) were purchased from the American Type Culture Collection.
2. T47D human breast cancer cells (HTB-133) were purchased from the American Type Culture Collection.

Authentication

Cell lines were authenticated by morphology and growth characteristics.

Mycoplasma contamination

Cells were negative for mycoplasma as verified with mycoplasma detection kit.

Commonly misidentified lines
(See ICLAC register)

No commonly misidentified cell lines were used.
